# Polymorphisms of Pyrimidine Pathway Enzymes Encoding Genes and HLA-B*40∶01 Carriage in Stavudine-Associated Lipodystrophy in HIV-Infected Patients

**DOI:** 10.1371/journal.pone.0067035

**Published:** 2013-06-26

**Authors:** Pere Domingo, Maria Gracia Mateo, Alain Pruvost, Ferran Torres, Juliana Salazar, Maria del Mar Gutierrez, M Carmen Cabeza, Joan Carles Domingo, Irene Fernandez, Francesc Villarroya, Francesc Vidal, Montserrat Baiget, Oscar de la Calle-Martín

**Affiliations:** 1 Infectious Diseases Unit, Hospital de la Santa Creu i Sant Pau, Universitat Autònoma de Barcelona, Barcelona, Spain; 2 CEA, iBiTecS, SPI, Laboratoire d’Etude du Métabolisme des Médicaments, Gif sur Yvette, France; 3 Biostatistics and Data Management Platform, IDIBAPS, (Hospital Clinic); Biostatistics Unit, School of Medicine, Universitat Autònoma de Barcelona, Barcelona, Spain; 4 CIBERER (U-705), Barcelona, Spain; 5 Department of Biochemistry and Molecular Biology, University of Barcelona, Barcelona, Spain; 6 Infectious Diseases and HIV/AIDS Unit, Department of Internal Medicine, Hospital Universitari Joan XXIII, IISPV, Universitat Rovira i Virgili, Tarragona, Spain; 7 Department of Genetics, Hospital de la Santa Creu i Sant Pau, Universitat Autònoma de Barcelona, Barcelona, Spain; 8 Department of Immunology, Hospital de la Santa Creu i Sant Pau, Universitat Autònoma de Barcelona, Barcelona, Spain; University of Cape Town, South Africa

## Abstract

**Purpose:**

To assess in a cohort of Caucasian patients exposed to stavudine (d4T) the association of polymorphisms in pyrimidine pathway enzymes and HLA-B*40∶01 carriage with HIV/Highly active antiretroviral therapy (HAART)-associated lipodystrophy syndrome (HALS).

**Methods:**

Three-hundred and thirty-six patients, 187 with HALS and 149 without HALS, and 72 uninfected subjects were recruited. The diagnosis of HALS was performed following the criteria of the Lipodystrophy Severity Grading Scale. Polymorphisms in the thymidylate synthase (TS) and methylene-tetrahydrofolate reductase (MTHFR) genes were determined by direct sequencing, HLA-B genotyping by PCR-SSOr Luminex Technology, and intracellular levels of stavudine triphosphate (d4T-TP) by a LC-MS/MS assay method.

**Results:**

HALS was associated with the presence of a low expression TS genotype polymorphism (64.7% vs. 42.9%, OR = 2.43; 95%CI: 1.53–3.88, P<0.0001). MTHFR gene polymorphisms and HLA-B*40∶01 carriage were not associated with HALS or d4T-TP intracellular levels. Low and high expression TS polymorphisms had different d4T-TP intracellular levels (25.60 vs. 13.60 fmol/10^6^ cells, P<0.0001). Independent factors associated with HALS were(OR [95%CI]: (a) Combined TS and MTHFR genotypes (p = 0.006, reference category (ref.): ‘A+A’; OR for ‘A+B’ vs. ref.: 1.39 [0.69–2.80]; OR for ‘B+A’ vs. ref.: 2.16 [1.22–3.83]; OR for ‘B+B’ vs. ref.: 3.13, 95%CI: 1.54–6.35), (b) maximum viral load ≥5 log10 (OR: 2.55, 95%CI: 1.56–4.14, P = 0.001), (c) use of EFV (1.10 [1.00–1.21], P = 0.008, per year of use).

**Conclusion:**

HALS is associated with combined low-expression TS and MTHFR associated with high activity polymorphisms but not with HLA-B*40∶01 carriage in Caucasian patients with long-term exposure to stavudine.

## Introduction

Despite similarities in antiretroviral drug exposure and similar immunological, virological and demographic characteristics, the variability in individual responses to antiretrovirals suggests the influence of host-dependent factors such as genetic make-up. Therefore, several pharmacogenetic studies have analyzed the influence of specific polymorphisms on the toxicity of antiretroviral treatment [1]. The analysis of pharmacogenetic determinants of toxicity has proved extremely successful with regard to the identification of the genetic basis of hypersensitivity reaction to abacavir and its causative link to HLA-B*57∶01 allele [2].

HALS is a long-term adverse effect limiting the doubtless efficacy of HAART, although its incidence has decreased since the use of thymidine analogues in general, and d4T in particular, has declined in developed countries [3]. The impact of HALS on patient quality of life, perceived stigma and psychologically devastating consequences may ultimately impact patient adherence to treatment, eventually leading to treatment failure [4]. The risk of developing changes in body fat distribution is variable despite similar HAART exposure, once again suggesting a genetic predisposition [1], [5]. Recently, the association of HLA-B*40∶01 with HALS has been reported in a South East Asian population treated with d4T [6]. We have showed the association of thymidylate synthase (TS) polymorphisms with HALS in a limited sample of patients [7], [8].

We undertook a study to confirm the possible association between HALS, TS and MTHFR polymorphisms and HLA-B*40∶01 carriage in a well-characterized cohort of Caucasian patients with long-term exposure to d4T. Furthermore, in a subset of these patients we determined d4T-TP intracellular levels in peripheral blood mononuclear cells.

## Methods

### Subjects

All subjects were recruited at the HIV infection clinic of the *Hospital de la Santa Creu i Sant Pau*, which attends a population of 1708 patients on active follow-up. They were patients with an established diagnosis of HIV infection on treatment. Patients were eligible if they had HALS and had been receiving therapy with d4T as part of their antiretroviral regimes. A normal dose of d4T was 40 mg and 30 mg twice daily if the patient weighed >60 kg or <60 kg, whereas a reduced dose was 30 mg/12 h and 20 mg/12 h for patients with a weight ≥60 or <60 kg, respectively. Subjects who were hospitalized or had a frank cognitive impairment such as delirium or dementia, opportunistic infections, neoplasms or fever of undetermined origin on enrolment were not eligible. At the time of study entry no patient used any other drug known to influence glucose metabolism or fat distribution such as anabolic hormones or systemic corticosteroids, uridine, recombinant human growth hormone, or appetite stimulants. Controls were patients followed in the same cohort who had not developed HALS despite having had d4T exposure that matched to that of patients (±6 months). Uninfected controls were healthy subjects without serologic evidence for HIV infection who were used for genetic comparisons. Both HIV-infected patients and controls were Caucasian subjects from the area of Barcelona, whose ethnicity could be traced to grandparents. The diagnosis of AIDS was based on the 1993 revised case definition of the Centers for Disease Control and Prevention [9]. Written informed consent was obtained from the patients at study entry. The study was approved by the Ethics Committee of the *Hospital de la Santa Creu i Sant Pau*.

### Body Composition Measurements

Subjects were weighed on calibrated scales after removing shoes, outdoor clothing, and other heavy items. Body mass index (BMI) was calculated by dividing the weight in kilograms by the square of the height in meters. Waist circumference was measured to the nearest millimeter using anatomical landmarks as defined for the Third National Health and Nutrition Evaluation Survey [10].

Whole body dual energy X-ray absorptiometry (DEXA) scans (Hologic QDR-4500A Hologic, Inc, 590 Lincoln St, Waltham, MA 02154, USA) were conducted by a single operator on all the patients. The percentage of fat at the arms, legs and central abdomen (calculated from the mass of fat versus lean and bone mass) as well as total lean body mass in kilograms was recorded.

### Definition of HALS and Metabolic Syndrome

Lipoatrophy was identified by patient self-reporting and/or clinician observation of relevant changes to the face (loss of cheek and/or preauricular fat pads), arms, legs, and subcutaneous abdominal tissue, and confirmed clinically by qualitative physical examination. Lipohypertrophy was diagnosed by the presence of central adiposity which was defined by a waist-hip ratio (WHR) of >0.90 in men, and >0.80 in women) [10], breast enlargement in women or the appearance of cervical fat pads or supraclavicular fat accumulation [11]. Both clinical situations occurred in an isolated manner or concomitantly and any combination of them was considered to be a mixed syndrome.

Visual aspects of lipodystrophy were assessed with a lipodystrophy severity grading scale (LSGS) based on that reported by Lichtenstein et al. [11]. The degree of lipoatrophy and diffuse fat accumulation at each region was rated as absent (score of 0), mild (noticeable on close inspection, score of 1), moderate (readily noticeable by patient/physician, score of 2), or severe (readily noticeable to a casual observer, score of 3). The overall score was the mean of the sum of the scores given by patient and physician both for fat loss or fat accumulation. A clinical diagnosis of lipodystrophy was given to a patient with an overall score >7. This score was chosen because it would be the LSGS of a patient with mild fat loss or mild fat accumulation at all the anatomical sites assessed (face, arms, buttocks, legs, abdomen, neck, breasts) [12]. Patients with severe fat changes in at least one body location were considered as lipodystrophic [12]. In addition, facial lipoatrophy was also assessed through the scale of Fontdevila et al. [13] which established four degrees of facial lipoatrophy ranging from normal to grade three (or severe).

The metabolic syndrome was defined according to the U.S. National Cholesterol Education Program (NCEP) Adult Treatment Panel III Guidelines [14] and modified as recommended in the latest American Heart Association/National Heart, Lung, and Blood Institute Scientific Statement [15].

### Biochemistry Laboratory Measurements

All laboratory investigations were performed as previously described [7].

### Measurement of Intracellular d4T Triphosphate Concentrations

d4T-TP was from Moravek Biochemicals (CA, USA) and Cl-ATP (2-chloroadenosine 5′-triphosphate) used as internal standard (IS) was from Sigma-Aldrich (St Quentin-Fallavier, France). In brief, after 2 blood samples (around 7 mL) were obtained, PBMCs were prepared by centrifugation with separation gradient medium (Ficoll Histopaque 1077, Sigma) and immediately stored at a temperature around −80°C pending analysis. The 2 samples were taken, the first 3 hours after dosing (to measure C_max_), and the second, 12 hours after dosing (C_through_). Our analysis, as pre-specified in the study protocol, was based on C_through_, and these concentrations are used throughout the manuscript. PBMCs were lysed in 1 ml of ice-cold methanol/Tris 0.05 M/HCl pH = 5; 70/30 (v/v) containing IS and, after methanol evaporation, a 40-mL fraction of the remaining solution was injected into the LC-MS/MS system.

The LC-MS/MS consisted of a LC-20A prominence liquid chromatograph (Shimadzu, Champs sur Marne, France) connected to a triple-quadruple mass spectrometer TSQ Quantum Discovery (Thermo Fisher Scientific, Les Ulis, France) operating in the negative ESI mode for the detection of both d4T-TP and IS [16]. The liquid chromatographic method was used according to a previously published method [17]. In these conditions, retention times were around 3.95 and 4.05 min. for d4T-TP and IS, respectively. Amount of d4T-TP was determined in the calibrated range from 50 to 3000 femtomoles (fmol) per cell pellet. Finally, the PBMCs of each clinical sample were counted using a validated biochemical test as previously described [18] in order to provide results in fmol/10^6 ^PBMC.

### Genotyping Analyses

The genomic DNA was extracted from the peripheral leucocytes by the salting-out procedure [19]. In the TS gene, the variable number tandem repeat (VNTR) of 28 bp polymorphism and the G>C SNP in the first and second repeat were analyzed. A DNA fragment was amplified using previously described PCR conditions and primers [7], and directly sequenced using an ABI PRISM 3100 Genetic Analyzer (Applied Biosystems, Foster City, CA, USA). This G to C substitution changes a critical residue in the USF E-box consensus element, abolishes USF-1 binding, and alters transcriptional activity. TS genotypes of the patients were classified according to Kawakami and Watanabe into two groups: high expression type (2/3G, 3C/3G and 3G/3G) and low expression type (2/2, 2/3C and 3C/3C) [20].

The MTHFR gene polymorphisms (677C→T [rs1801133] and 1298 A→C [rs1801131) were determined. These two polymorphisms were analyzed using Fluidigm’s Biomark system. This technology is designed for the allelic discrimination 5′ nuclease assay. The samples and the TaqMan Gene expression assays (Applied Biosystems, Foster City, CA, USA) were prepared following manufacturer’s instructions. The 48.48 dynamic arrays used were automatically loaded using an IFC Controller (Fluidigm Corporation), and real-time reactions were performed and analyzed using BioMark Real-Time PCR System and Analysis software (Fluidigm Corporation), respectively. As a quality control, normal, heterozygote and homozygote sequenced samples were included on every array for each genotype. MTHFR genotypes were classified also into two groups: those associated with a decreased enzymatic activity (homozygous 677T, homozygous 1298C and compound heterozygous patients), and genotypes associated with an increased enzymatic activity (heterozygous and wild-type patients) [21].

HLA-B genotyping was performed by PCR-SSOr Luminex Technology. HLA-B40+ patients were further analyzed by sequencing of exons 2, 3 and 4 of the HLA-B locus.

### Statistical Analyses

Data are expressed as median with interquartile range (IQR) or as frequencies and percentages or otherwise specified. Continuous variables were assessed with the Mann-Whitney non-parametric test. Categorical data such as genotype and allele frequencies were compared by use of the Fisher’s exact test. A logistic regression analysis was used to examine the association of HALS with TS polymorphisms and other parameters; variables associated with a P<0.1 in the univariate analyses were included in the multivariate stepwise analysis. All analyses were performed with the SAS version 9.2 software (SAS Institute Inc., Cary, NC). The level of significance was established at 0.05 and all reported P values are two-sided.

## Results

### Population Studied

We studied 336 HIV-infected, Caucasian patients, exposed to d4T-based regimes. Demographic, means of acquiring HIV infection and the virological and immunological status of patients with and without HALS are shown in table 1. There were 255 men (75.9%) and 81 women (24.1%), with a mean age of 46.5±9.1 years (median: 44.0 [IQR: 41.0–50.0 years]). One hundred and thirty-five patients (40.2%) had had a prior AIDS-defining condition. No patient was diabetic or was using insulin or hypoglycemic agents. Seventy-two uninfected controls were also studied for genetic markers.

**Table 1:** Demographics and viro-immunological status of the population studied.

Parameter	HALS (n = 187)	No HALS (n = 149)	P value
Age, years	44.0 (40.0–51.7)	44.0 (41.0–49.0)	0.8171
Men, n (%)	137 (73.3)	118 (79.1)	0.2495
Means of HIV-1 infection			0.2538
MsM, n (%)	53 (28.3)	54 (36.2)	
HTSX, n (%)	77 (41.2)	51 (34.2)	
IDU, n (%)	57 (30.5)	44 (29.5)	
Years since diagnosis	13.0 (9.0–17.0)	13.0 (10.0–15.2)	0.9607
Smokers, n (%)	89 (47.6)	67 (44.9)	0.6699
Alcohol abuse, n (%)	12 (6.4)	14 (9.4)	0.4261
Prior AIDS, n (%)	85 (45.4)	50 (33.5)	0.0344
HCV co-infection, n(%)	69 (36.9)	58 (38.9)	0.7323
HBV co-infection, n (%)	23 (12.3)	21 (14.1)	0.7425
Current CD4 count, cells/mm^3^	551 (387–835)	583 (403–812)	0.4862
CD4 increase, cells/mm^3^	346 (197–605)	340 (207–518)	0.5254
Current CD8 count cells/mm^3^	835 (530–1125)	864 (626–1186)	0.4546
CD8 increase, cells/mm^3^	310 (106–585)	281 (106–602)	0.8508
Nadir CD4 cell count <100 cells/mm^3^, n (%)	78 (41.7)	43 (28.8)	0.0164
Nadir CD4 cell count <200 cells/mm^3,^ n (%)	112 (59.9)	70 (46.9)	0.0211
Current viral load, log_10_, copies/ml	1.28 (1.28–1.28)	1.28 (1.28–1.67)	0.3001
Undetectable viral load, n (%)	141 (75.4)	108 (72.5)	0.6163
Maximum viral load ≥5 log_10_, copies/ml	130 (69.5)	77 (51.7)	0.0010
Viral load decrease, log_10_, copies/ml	−3.72 (−2.94 [−4.35])	−3.68 (−2.60 [−4.26])	0.4278

Values are expressed as median and interquartile range, unless indicated. HALS = HIV-1/HAART-associated lipodystrophy syndrome, MsM = men who have sex with men, HTSX = heterosexuals, IDU = intravenous drug users, AIDS = acquired immune deficiency syndrome, HCV = hepatitis C virus, HBV = hepatitis B virus, ml = millilitres.

### Antiretroviral Drug Exposure and Immuno-virological Status

The immuno-virological status of patients and controls and their cumulated exposure to antiretroviral drugs is shown in tables 1 and 2. Both groups were well balanced except for prior AIDS (OR = 1.65; 95%CI: 1.03–2.64, P = 0.0359), a CD4 cell nadir <200 cells/mm^3^ (OR = 1.69; 95%CI: 1.07–2.66, P = 0.0244), and a higher baseline viral load ≥5 log_10_ (OR = 2.13; 95%CI: 1.33–3.42, P = 0.0012) more frequent among HALS patients, as expected. Eighty-two patients of the HALS group (43.8%) and 71 patients (47.6%) of the non-HALS group were receiving reduced d4T doses (OR = 0.86; 95%CI: 0.54–1.35, P = 0.5587). Exposure to thymidine analogues, specifically d4T, was comparable between groups (table 2).

**Table 2:** Antiretroviral drug exposure in the population studied.

Parameter	HALS (n = 187)	No HALS (n = 149)	P value
Current ART composition			0.0660
PI-based, n (%)	82 (43.8)	72 (48.3)	
NNRTI-based, n (%)	98 (52.4)	69 (46.3)	
3 NRTI, n (%)	7 (3.7)	8 (5.4)	
Current d4T use, n (%)	45 (19.2)	30 (14.7)	
Current AZT use, n (%)	17 (9.1)	23 (15.4)	0.0900
Individual drug exposure*			
3TC/FTC exposure (m)	56.0 (26.2–95.5)	51.0 (20.0–80.0)	0.0830
ddI exposure (m)	29.0 (0.0–66.7)	12.0 (0.0–48.0)	0.0139
EFV exposure (m)	6.0 (0.0–0−51.0)	0.0 (0.0–28.0)	0.0095

All parameters expressed as median and (interquartile range) unless indicated. HALS = HIV-1/HAART-associated lipodystrophy syndrome, ART = antiretroviral therapy, PI = protease inhibitor, NNRTI = non-nucleoside reverse transcriptase inhibitor, NRTI = nucleoside reverse transcriptase inhibitor. *There were not individual druga exposure differences (P<0.1) for stavudine, tenofovir, zidovudine, zalcitabine, abacavir, and nevirapine, m = months, 3TC = lamivudine, FTC = emtricitabine, ddI = didanosine, EFV = efavirenz, m = months.

### Diagnosis of HALS and Metabolic Syndrome

All 187 cases with HALS presented with lipoatrophy, but in addition 100 (53.5%) had a mixed lipoatrophic-lipohypertrophic phenotype. The anthropometric data of patients with and without HALS are shown in table 3. One hundred and forty-one patients (41.9%) had metabolic syndrome. Metabolic syndrome was associated with the presence of HALS (47.1% vs. 35.6%, OR = 1.61; 95%CI: 1.01.2.57, P = 0.0446) and with carriage of the HLA-B*40∶01 genotype (7.8% vs. 2.1%, OR = 4.04; 95%CI: 1.16–17.71, P = 0.0244).

**Table 3:** Anthropometric, metabolic and fat data in d4T-exposed patients.

	HALS (n = 187)	No HALS (n = 149)	P value
Weight, kg	66.5 (58.0–73.0)	70.0 (61.5–75.0)	0.2293
BMI, kg/m^2^	23.4 (21.2–25.5)	23.4 (21.1–25.3)	0.9218
Waist circumference, cm	87.0 (81.0–94.0)	88.0 (82.0–94.0)	0.8412
WHR	0.94 (0.90–1.01)	0.93 (0.88–0.97)	0.0109
LSGS score, units	9.0 (5.0–11.5)	2.0 (0.5–5.0)	<0.0001
Facial score, units	2.0 (1.0–3.0)	0 (0.0–1.0)	<0.0001
Systolic BP, mm Hg	120 (110–130)	114 (108–130)	0.0034
Diastolic BP, mm Hg	75 (70–80)	75 (70–80)	0.7552
Metabolic syndrome, n (%)	88 (47.1)	53 (35.6)	0.0446
Total cholesterol, mmol/l	5.05 (4.16–5.93)	4.95 (4.25–5.65)	0.3669
Triglycerides, mmol/l	1.92 (1.18–3.18)	1.63 (1.09–2.50)	0.0171
HDL cholesterol, mmol/l	1.09 (0.91–1.35)	1.18 (0.99–1.49)	0.0047
LDL cholesterol, mmol/l	3.12 (2.49–3.87)	2.77 (2.22–3.38)	0.0007
VLDL cholesterol, mmol/l	0.89 (0.55–1.31)	0.73 (0.48–1.06)	0.0035
Fasting glucose, mmol/l	5.4 (4.9–5.9)	5.2 (4.9–5.6)	0.0220
Fasting insulin, pmol/l	80.0 (55.0–122.2)	61.0 (36.7–86.0)	<0.0001
HOMA-IR	2.81 (1.85–4.34)	2.00 (1.13–2.99)	<0.0001
Total body fat, g	12012 (8589–15733)	14089 (10783–19586)	0.0001
Total body fat, %	18.1 (14.4–23.9)	22.5 (17.1–27.1)	0.0002
Trunk fat, g	7368 (5573–10550)	7920 (5718–10761)	0.6327
Appendicular fat, g	3180 (2026–5126)	4275 (2924–6809)	<0.0001
Trunk/appendicular fat ratio	2.25 (1.68–3.01)	1.64 (1.15–2.37)	<0.0001

All values expressed as median and (interquartile range) unless specified.

HALS = HIV-1/HAART-associated lipodystrophy syndrome, BMI = body mass index, WHR = waist-hip ratio, LSGS = lipodystrophy grade severity score, BP = blood pressure, mmol/l = milimoles per litre, HDL = high density lipoprotein, LDL = low density lipoprotein, VLDL = very low density lipoprotein, pmol/l = picomoles per litre, HOMA-r = homeostasis model assessment, g = grams.

### HLA-B*40∶01 Carriage and TS and MTFHR Genotypes

One hundred and eighty-five patients (55.1%) had a low expression TS genotype whereas 15 patients were positive for HLA-B*40∶01 (table 4). Low expression TS genotype was more frequent in patients with HALS than in patients without (64.7% vs. 42.9%, OR = 2.43; 95%CI: 1.53–3.88, P<0.0001). There were not statistically significant differences between HALS and non-HALS patients with respect to MTHFR 677C→T (P = 0.9282) or MTHFR 1298A→C genotypes (P = 0.9612) neither there were for HLA-B*40∶01 carriage (P = 0.7983). MTHFR genotypes with increased enzymatic activity were not more frequent in cases than in controls (63.1% vs. 66.4%, OR = 0.86, 95%CI: 0.54–1.39, P = 0.6020). When TS and MTHFR genotypes were combined, TS genotypes of low expression plus high enzymatic activity MTHFR genotypes were the most frequent in HALS patients (Table 4). However, when comparing low expression TS genotypes with respect to MTHFR genotype there were not differences between those with increased or decreased enzyme activity (OR = 0.82; 95%CI. 0.41–1.64; P = 0.6782).

**Table 4:** Gene polymorphisms associated with the presence of HALS in d4T-exposed patients.

	HALS (n = 187)	No HALS (n = 149)	P value
2R/2R, n (%)	46 (24.6)	17 (11.4)	Not tested
2R/3C, n (%)	53 (28.3)	33 (22.1)	
2R/3G, n (%)	33 (17.7)	40 (26.9)	
3C/3C, n (%)	22 (11.8)	14 (9.4)	
3C/3G, n (%)	24 (12.8)	36 (24.2)	
3G/3G, n (%)	9 (4.8)	9 (6.0)	
Low expression, n (%)	121 (64.7)	64 (42.9)	<0.0001
High expression, n (%)	66 (35.3)	85 (57.1)	
C/C, n (%)	70 (37.4)	56 (37.6)	0.9282
C/T, n (%)	85 (45.4)	70 (46.9)	
T/T, n (%)	32 (17.1)	23 (15.4)	
A/A, n (%)	98 (52.4)	78 (52.3)	0.9612
A/C, n (%)	72 (38.5)	56 (37.6)	
C/C, n (%)	17 (9.1)	15 (10.1)	
Genotypes with increased activity*, n (%)	118 (63.1)	99 (66.4)	0.6020
Genotypes with decreased activity†, n (%)	69 (36.9)	50 (33.5)	
Low expression and increased activity (B+A), n (%)	78 (41.7)	44 (29.5)	0.0009
Low expression and decreased activity (B+B), n (%)	43 (22.9)	20 (13.4)	
High expression and increased activity (A+A), n (%)	40 (21.4)	55 (36.9)	
High expression and decreased activity (A+B), n (%)	26 (13.9)	30 (20.1)	
Positive, n (%)	9 (4.8)	6 (4.0)	0.7983
Negative, n (%)	176 (93.1)	143 (96.0)	

HALS = HIV/HAART-associated lipodystrophy syndrome, TS = thymidylate synthase, MTHFR = methylene-tetrahydrofolate reductase,

* Heterozygous and wild-type patients,

† Homozygous 677T, homozygous 1298C and compound heterozygous patients.

Nine HALS patients (4.5%) and 4 uninfected controls (5.5%) were found to be positive for HLA-B*40∶01 (OR = 0.86, 95%CI: 0.23–3.95, P = 0.7589). There were significant differences between these groups with respect to TS polymorphisms (OR = 2.29; 95%CI: 1.27–4.14, P = 0.0046), but not with respect to MTHFR 677C→T (P = 0.0940) or MTHFR 1298A→C (P = 0.1080) polymorphisms.

There were not significant differences between non-HALS patients and uninfected controls with respect to TS (OR = 0.94; 95%CI: 0.51–1.73, P = 0.9482), MTHFR 677C→T (P = 0.0940) or MTHFR 1298A→C (P = 0.1080) polymorphisms (P = 0.1180 and P = 0.0950) or with respect to HLA-B*40∶01 carriage (P = 0.7315).

### d4T-TP Intracellular Levels and Genotypes

d4T-TP intracellular concentrations were determined in 41 HALS and 45 non-HALS patients, including those positive for HLA-B*40∶01. The demographic, anthropometric, fat distribution and antiretroviral drug exposure of these patients were not significantly different from those of the whole study groups. The median d4T-TP concentration in the whole cohort was 18.30 fmol/10^6^ cells (interquartile range [IQR]: 11.00–28.50 fmol/10^6^ cells). The median concentration of d4T-TP in patients treated or not treated with EFV were 19.70 (IQR: 13.80–26.50) and 16.85 (10.30–30.0) fmol/10^6^ cells, respectively (P = 0.5659). The median concentrations of d4T-TP for patients with or without HALS were 26.70 (IQR: 20.50–54.0) and 13.60 (IQR: 7.60–18.00) fmol/10^6^ cells (P<0.0001), respectively. Intracellular levels of d4T-TP by genotypes and combinations thereof are shown in table 5.

**Table 5:** d4T-TP intracellular levels according to TS and MTHFR genotypes, and HLA B*-40∶01 carriage.

Genotype	d4T-TP, fmol/10^6^ cells	P value
**Thymidylate synthase**		
Low expression	25.6 (18.60–33.0)	<0.0001
High expression	13.60 (7.00–20.10)	
**MTHFR genotypes and enzymatic activity**		
Genotypes with increased activity*, n = 59	19.40 (11.12–29.17)	0.7236
Genotypes with decreased activity†, n = 27	14.80 (11.39–26.90)	
**Combined TS and MTHFR genotypes**		
Low expression and increased activity (B+A), n = 29	24.80 (19.27–33.50)	<0.0001
Low expression and decreased activity (B+B), n = 12	26.90 (13.90–33.60)	
High expression and increased activity (A+A), n = 30	13.50 (6.90–20.10)	
High expression and decreased activity (A+B), n = 15	13.6 (10.52–26.50)	
**HLA-B** * **4001 genotype**		
Positive, n = 15	20.20 (12.90–26.85)	0.7847
Negative, n = 71	16.60 (11.00–29.17)	

Values are expressed as median (interquartile range) unless otherwise indicated. TS = thymidylate synthase, MTHFR = methylene-tetrahydrofolate reductase,

* Heterozygous and wild-type patients,

† Homozygous 677T, homozygous 1298C and compound heterozygous patients.

### Independent Predictors of Development of HALS

When excluding TS genotype, the predictors were (OR [95%CI]: (a) Combined TS and MTHFR genotypes (p = 0.006, reference category (ref.): ‘A+A’; OR for ‘A+B’ vs. ref.: 1.39 [0.69–2.80]; OR for ‘B+A’ vs. ref.: 2.16 [1.22–3.83]; OR for ‘B+B’ vs. ref.: 3.13, 95%CI: 1.54–6.35), (b) maximum viral load ≥5 log10 (OR: 2.55, 95%CI: 1.56–4.14, P = 0.001), (c) use of EFV (1.10 [1.00–1.21], P = 0.008, per year of use) and (c) use of ddI (1.12 [1.03–1.22], P = 0.053, per year of use); of note, the latter was very close to but did not reach statistical significance.

## Discussion

Our study does not show an association between the carriage of HLA-B*40∶01 and HALS in Caucasian patients, unlike what was found in Thai patients [6], whereas it confirms the previous found association of low expression TS polymorphisms, especially when combined with MTHFR polymorphisms associated with high enzyme activity, with the development of HALS [8]. This is probably related to the fact that low expression TS polymorphisms are associated with increased d4T-TP intracellular levels, which in turn have also been associated with the development of HALS [8]. However, we could not find a correlation between the presence of HLA-B*40∶01 either with HALS or with d4T-TP intracellular levels whereas we could find a contribution of MTHFR genotypes to increased d4T-TP intracellular levels. Therefore, our results suggest that carriage of HLA-B*40∶01 is neither a risk for HALS or for high intracellular levels of d4T-TP in Caucasian patients on a d4T-based antiretroviral regime.

However, our results may have inherent limitations. This is a cross-sectional study and, therefore no causal relationships can or must be drawn. The prevalence of percentage of HLA-B*40∶01 genotype carriage among Thai patients with HALS was 29% whereas it was only 4.8% among our patients [6]. However, the frequency of HLA-B*40∶01 carriage among our patients is similar to that found in uninfected Caucasian Spaniards controls from the same geographic location. Furthermore, the frequency of HLA-B*40∶01 carriage among uninfected controls (5.5%) lies within the range found for other Spanish populations and for population from neighboring areas [22].

TS, a key enzyme in *de novo* synthesis of pyrimidines, has been shown to modulate d4T-TP intracellular levels through a VNTR polymorphism in the TS promoter region and a single nucleotide polymorphism (a common G>C change in the second repeat of the alleles containing three repeats), both having functional translation [8]. We have confirmed that polymorphisms of TS linked to a low expression of the enzyme are associated with increased d4T-TP levels which may have consequences in terms of toxicity [7], [8]. The different genotypes of the VNTR in the TS promoter region occur at different frequencies in different populations. In Caucasians, heterozygosity occurs in approximately 50% of the population, and each of the two homozygous genotypes is found in approximately 25% of the population [23], [24]. The same is true for black people and for Southwest Asians [23], [24]. However, homozygous triple repeat subjects were twice as common in Chinese subjects (67%) as in Caucasian subjects (38%) [23]. This may explain why HALS, a toxicity that has been directly linked to thymidine analogues, which have been widely used all over the world, has been so rarely reported in Far East Asian countries [25]. A similar phenomenon, i.e. an ethnic distribution of HLA-B*40∶01 would be a possible explanation of the discrepant findings between the Thai study and ours. The HLA-B*40∶01 allele frequency is 0.0226 in our HIV population, in a sample of 3292 healthy patients from our Hospital and from a blood donor center in Madrid was 0.0224 (unpublished data), whereas in Thai population the HLA-B*40∶01 allele frequency was 0.0550 [22]. The prevalence difference between HALS and non-HALS patients found in our study was 0.84%. Therefore, our results may rule out differences from −4.19% to +5.49%. If a study is to be undertaken in a population similar to ours, then for differences ranging from 1% to 5% would imply recruiting samples ranging from 381 to 6745 individuals per group [26], [27]. Recently, an Australian study could not identify any association of HLA-B*40∶01 genotype with thymidine analogues-induced peripheral fat loss in Caucasians [28]. In this study, carrying HLA A01, B08 and DQ2 supertype alleles protected patients from thymidine analogues-associated peripheral fat [28].

Another possible explanation could be that HLA-B*4001 is in linkage disequilibrium with another close gene, i.e. the tumor necrosis alpha (TNF-α gene) [29]. If this was the case, then the inflammatory state would promote the over-expression of nucleoside transporters [30], leading to increased d4T-TP intracellular levels. However, our study does not suggest any link between HLA-B*40∶01 carriage and d4T-TP intracellular levels. A number of studies have assessed the effect of different polymorphisms in genes encoding for mitochondrial DNA [1], [5], lamin A [1], [5], TNF alpha [1], [31] and interleukins 1 beta and 6 [1], [31], [32] in the pathogenesis of HALS. However, none of these reports had conclusive results and most of them could not have been replicated [32]. In fact, a recent meta-analysis on the role of polymorphisms in the TNF alpha promoter gene concluded that there is no association with the risk of developing HALS [33].

MTHFR polymorphisms have also an impact on enzyme activity, which in turn may have consequences on TS activity through formation of a ternary inhibitory complex with TS, 5, 10-methylene tetrahydrofolate and deoxyuridine monophosphate [21]. Therefore, they could theoretically contribute both to increased d4T-TP levels and HALS. Our results suggest that MTHFR polymorphisms have a marginal contribution both to increased d4T-TP levels and HALS.

The link between EFV exposure and the appearance of HALS has been intensely debated in latest years due to controversial evidence [34]. We have found EFV exposure to be an independent predictor of HALS in our d4T-exposed population. However, EFV implication in HALS pathogenesis has not been proved in studies avoiding thymidine analogues [35], [36]. We did not find different d4T-TP intracellular levels with respect to EFV exposure. Therefore, if EFV is involved, the pathogenic mechanism is not through increasing d4T-TP intracellular levels.

In summary, our results suggest that HLA-B*40∶01 carriage is not, whereas TS and MTHFR polymorphisms are, associated with HALS in Caucasian HIV-infected patients with long-term exposure to d4T-based antiretroviral regimes.
